# Machine Learning Inspired Nanowire Classification Method based on Nanowire Array Scanning Electron Microscope Images

**DOI:** 10.12688/openreseurope.16696.2

**Published:** 2024-06-28

**Authors:** Enrico Brugnolotto, Preslav Aleksandrov, Marilyne Sousa, Vihar Georgiev

**Affiliations:** 1 James Watt School of Engineering, University of Glasgow, Glasgow, Scotland, UK; 2 IBM Research Europe - Zurich, Rüschlikon, Säumerstrasse 4, 8803, Switzerland

**Keywords:** computer vision, nano-materials, nano- wires, image segmentation, machine learning, microscopy imaging, scanning electron microscopy

## Abstract

**Background:**

This article introduces an innovative classification methodology to identify nanowires within scanning electron microscope images.

**Methods:**

Our approach employs advanced image manipulation techniques in conjunction with machine learning-based recognition algorithms. The effectiveness of our proposed method is demonstrated through its application to the categorization of scanning electron microscopy images depicting nanowires arrays.

**Results:**

The method’s capability to isolate and distinguish individual nanowires within an array is the primary factor in the observed accuracy. The foundational data set for model training comprises scanning electron microscopy images featuring 240 III-V nanowire arrays grown with metal organic chemical vapor deposition on silicon substrates. Each of these arrays consists of 66 nanowires. The results underscore the model’s proficiency in discerning distinct wire configurations and detecting parasitic crystals. Our approach yields an average F1 score of 0.91, indicating high precision and recall.

**Conclusions:**

Such a high level of performance and accuracy of ML methods demonstrate the viability of our technique not only for academic but also for practical commercial implementation and usage.

## Introduction

The size of current transistors is in the range of tens of nanometers, and such ultra-scaled dimensions pose many fabrication challenges ^1, 2^. One challenge is finding the best way to connect the transistors, as in the best interconnect material and mode. The current industrial standard is to fabricate the interconnect using many conducting layers of metals and their alloys ( *e.g.* Cu and Ru) ^3, 4^. However, metal interconnects offer their own challenges at such small scales such as being bound by the aspect ratio limit in bit rate capacity and cross talk between different electrical lines ^5^.

To overcome some of these issues, there is growing interest in substituting metal with miniaturized active photonic components for short-range optical interconnects ^5– 7^. Materials such as III-V semiconductors are known for their superior light adsorption and emission qualities ^8, 9^. They are currently integrated on silicon (Si) substrates with pick-and-place ^10^ or wafer bonding methods ^11^. This allows manufacturers to avoid defects related to lattice mismatch but limits integration density and alignment precision ^12, 13^. Monolithic growth of III-V semiconductors on Si can achieve quick, high-precision, simultaneous integration on large wafer scales.

Template assisted selective epitaxy (TASE) allows high defect control when growing III-V nano- and microstructures monolithically on silicon ^14, 15^. Analysis of the evolution of the growth front in TASE-grown heterostructured III-V nanowires is fundamental to ensure homogeneous heterolayer thickness ^16^ and composition, in the case of ternary III-V compounds ^17^. Achieving high uniformity across the entire wafer surface is critical, as these factors affect the optical behavior of the device.

Heterostructured III-V micro- and nanostructures are usually analyzed using scanning transmission electron microscopy (STEM) to determine the evolution and stability of the growth front ^18, 19^, as well as the presence of crystalline defects ^20, 21^. STEM analysis has shown ^19^ that stabilization of the growth front tends to occur early in the growth process. This stabilisation improves the growth rate and geometry uniformity in TASE grown III-V nanowire. As a result, contact design is simplified, compared to previous experimental results where the metallic deposition area had to be defined on a wire by wide basis ^16^. However, sample preparation for STEM is long and destructive ^22^, making scanning electron microscopy (SEM) preferable for yield and reproducibility studies.

For the samples presented in this work, we selected growth conditions that stabilize a single {1 1 1} facet as a growth front perpendicular to the wafer surface for nanowires containing heterolayers of InP and InGaAs grown directly from a silicon seed ^19, 23^. From this knowledge, it is already possible to classify the wires as perfect from an SEM top-down view of the wire if the seed facet and the end facet are parallel along the growth direction and as defective otherwise.

When growing densely integrated submicrometer-sized III-V crystals, the current approach of manually cataloguing different growth outcomes on even a 2 cm *×* 2 cm chip becomes a very time-consuming and tedious process. One way to decrease the time requirements of such a study is to use machine learning (ML) methods. ML is a powerful tool for handling large amounts of data and has found application in materials science for various tasks. Classification ^24, 25^ and segmentation ^20^ of electron microscopy images can be used to deduce device properties, if the images contain sufficient information ^26^. ML models for identifying different types of nanostructures, such as the difference between nanowires, nanoparticles, and nanoporous substrates, have been shown to have extremely high accuracy ^27^. However, models that are tasked with segmentation often do not perform as well ^28^.

Classifying images containing multiple objects with fine differences is complex because the variability between nanowire images is of the same magnitude and quality as the target imperfections. Additionally, SEM images contain a high degree of noise that impedes the performance of traditional classification methods ^29^. Due to the large number of images, this process can benefit from automatization ^30^, especially when the need to use a less resolved and time-consuming characterization method arises ^31^.

Hence, to tackle some of the abovementioned problems, we describe a novel ML-based approach in this paper. For the purpose of this work, we introduce and characterize a new dataset for nanowire classification. Finally, we evaluate the performance of our method on the aforementioned dataset and discuss future research areas.

This article makes the following contributions to the current state of the art:

1)   To the best of our knowledge, this work shows the first kernel-based splitting algorithm for III-V nanowire array SEM images.

2)   Our work provides a new nanowire classification dataset containing annotated images of III-V nanowire arrays.

3)   In this work we demonstrate the performance of a compact convolutional neural network (CNN) classifier on the aforementioned dataset.

## Methods

**Figure 1.**
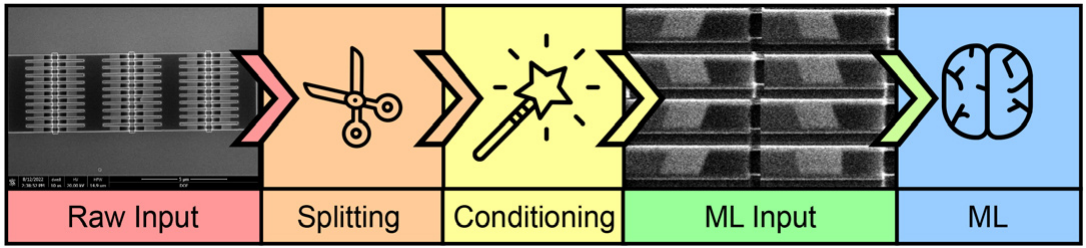
Process flow of classification method. The raw input consists of scanning electron microscope (SEM) images divided into individual wires through a splitting algorithm. The wire images are conditioned and later used as the input for a convolution neural network (CNN) classifier in our machine learning (ML) method.

To extract individual nanowires for classification from each SEM image, we developed a novel algorithm that finds the location of each nanowire and extracts it ^32^. The following algorithms were written in Python 3.9 ^33^, using OpenCV ^34, 35^ as the image manipulation package. The algorithm locates the nanowires and is based on two kernel operations applied to an image. These operations minimize the effects of noise and enhance wire edges. The kernel manipulations used are similar to, but distinct from, the typical definition used in machine learning. In this approach, the kernel operates *in situ* and transforms an image into a filter to locate feature edges. Once the edges are located, we split the image into granular blocks and condition each image through histogram equalization. This produces the final input to the ML model. The entire pipeline of operation is shown in Figure 1.

### A. Kernel operations

**Figure 2.**
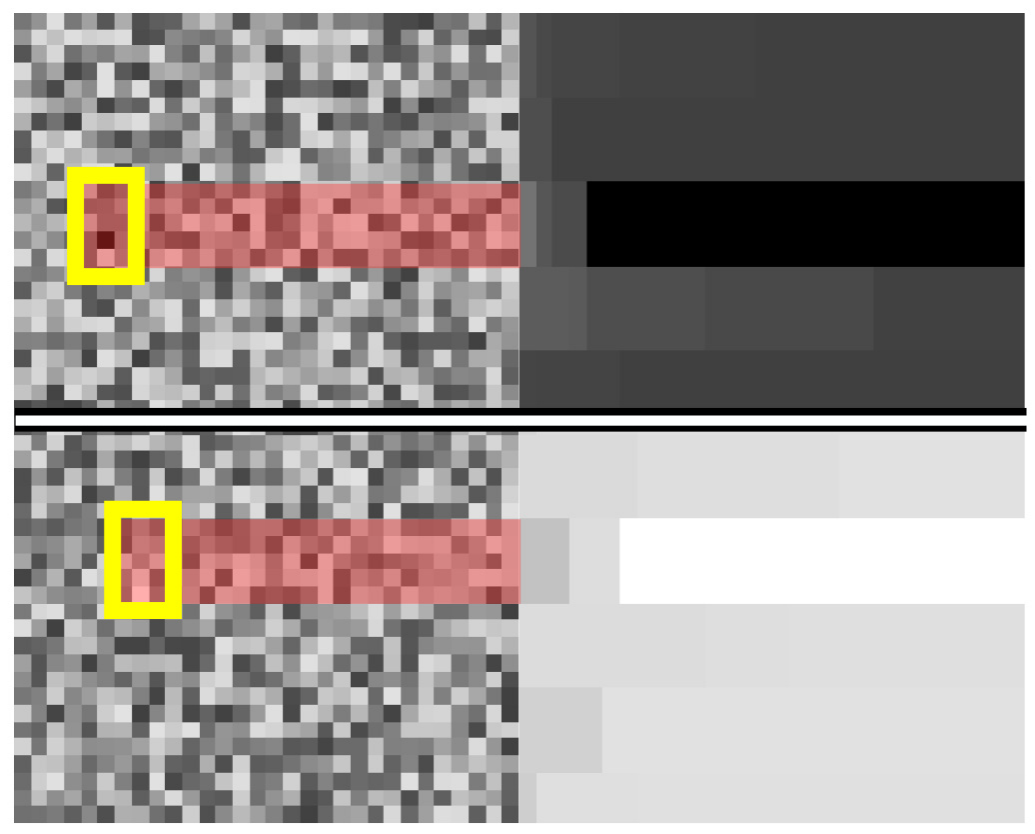
Mode of operation of kernel min and kernel max. The application of the MinKernel is shown on the top row. The raw input from the left is transformed into the image on the right. The section with the darkest pixel value is highlighted. The application of the MaxKernel is shown on the bottom row. The raw input from the left is transformed into the image on the right. The section with the lightest pixel value is highlighted.

As already discussed, our algorithm is based on two kernel operations. Both kernel operations are functions which act similarly. Each kernel searches for an extremum in a section of the image and then overwrites the whole section with the extremum value. Afterwards, the kernel moves along its row, ensuring an overlap between the previous and current window. At the end of a row, the kernel moves down the image and repeats the process without vertical overlap. This creates hard edges in the SEM image, removing any noise. Therefore, these operations find the rolling extremum in a row and propagate it forward. The two kernels find the minimum and maximum and are termed MinKernel and MaxKernel, respectively. The effect of these kernels on a noisy image is shown in Figure 2. There, it can be seen that the pixel values with the most extreme values are applied to the entire kernel and, due to the overlap, are extended to the end of the image.

### Algorithm

The algorithm splits up an image of an array, as in Figure 4, into its constituent nanowires by combining rotation, cut, split, and mirror operations. To simplify the nanowire isolation process, we have improved the cut operation to detect feature edges to cut around automatically. The operation is performed using information derived from MinKernel and MaxKernel. We devised a smart image paradigm that allows us to map a subsection of the visual domain onto its original coordinates and ultimately apply the cut on the original image for extraction. In this way, we can freely perform modifications, and once we determine the correct boundary, we can map back onto the original image and extract the nanowire picture. MinKernel and MaxKernel are used with gradient methods to identify where the cut point should be placed. The complete cut algorithm is shown in Algorithm 1.

**Algorithm 1. Cut algorithm**

**Require:** MinKernel

**Require:** MaxKernel

**Require:** Split(image, location or number of outputs)

**Require:** *img* - input image

    images are treated as 2D arrays following right hand coordinate system

    
**for** each side **do**

          minImg ← MinKernel( *img*)

          maxImg ← MaxKernel( *img*)

          
**for** each *col* in maxImg **do**

                maxV ← max( *col*)

          
**end for**

          
**for** each *col* in minImg **do**

                minV ← min( *col*)

          
**end for**

          maxGrad ← $\left|\frac{\partial \;maxV}{\partial x}\right|$

          minGrad ← $\left|\frac{\partial \;minV}{\partial x}\right|$

          cutPoint1 ← arg max _*x*_( *maxGrad*)

          cutPoint2 ← arg max _*x*_( *minGrad*)

          
*img* ← Split( *img*, min(cutPoint1, cutPoint2))[1]

          rotate *img* by 90°

    
**end for**

    
**return** *img*

The full algorithm used to isolate a nanowire is shown in Algorithm 2. It involves cutting and rotating the image to isolate the main three-column array. Afterwards, one can split the array symmetrically into six parts. Then, each part is split again into eleven equally spaced rows. The base images are not perfectly aligned with the horizontal axis. Regardless, the algorithm works effectively for small degrees of tilt, up to 5°. For larger tilts, the methodology fails; however, as the environment is heavily controlled, the tilt in the images is significantly limited. Thus making our methodology suitable for use. Additionally, the rotation should not affect the classification accuracy since a wire’s features, which determine its class, are rotation-independent up to some allowable limit. The main features are how parallel the wire fronts are and the presence of a parasitic crystal growing on the surface of the wire.

**Algorithm 2. Process algorithm**

**Require:** MinKernel

**Require:** MaxKernel

**Require:** Cut(image)

**Require:** Split(image, location or number of outputs)

**Require:** *img* - input image of nanowire array

    
*img* ← Cut( *img*)

    
*imgs* ← Split( *img*, 6 columns)

    
*wires* ← empty list of nanowire images

    
**for** *i* in *imgs* **do**

         
*i* ← Cut( *i*)

         
*tmpWires* ← Split( *i*, 11 rows)

         
**for** *j* in *tmpWires* **do**

              
*tmpWire* ← Cut( *j*)

              
*tmpWire* ← Histogram equalization of *tmpWire*

              Append *tmpWire* to *wires*

         
**end for**

     
**end for**

     
**return** *wires*

### Machine learning model

**Figure 3.**
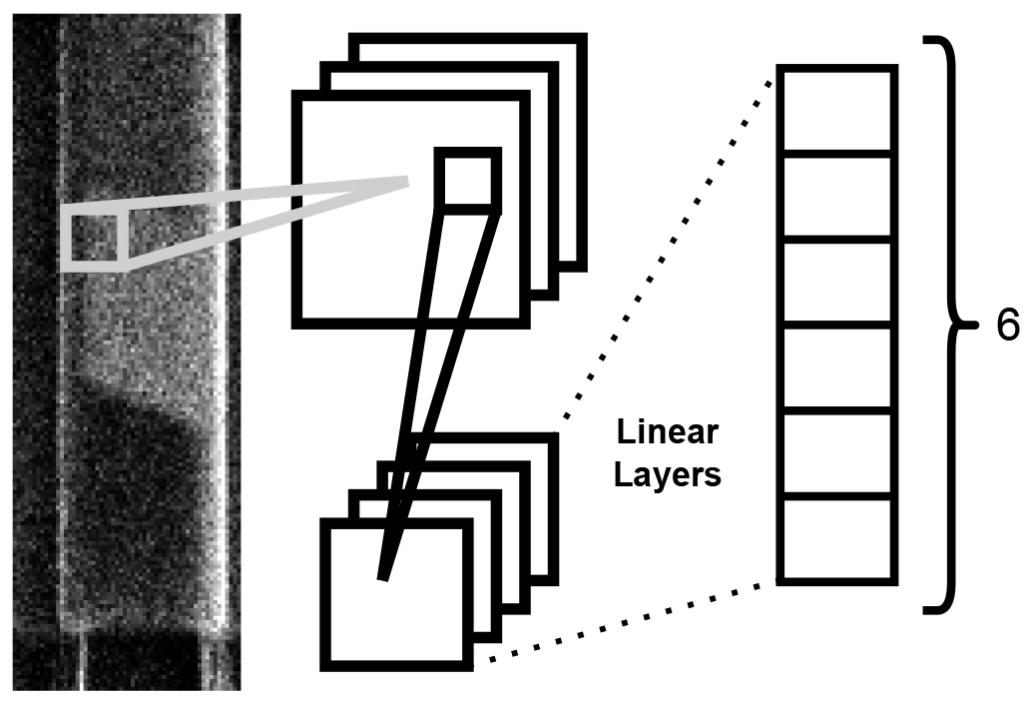
Diagram of the operation of the ML model used for nanowire classification. Each nanowire image is fed through to two convolutional layers followed by three linear layers producing a final output of six probabilities.

The primary objective of this research paper is categorizing diverse visual representations depicting heterostructured nanowires. As previously mentioned, the pivotal impediment within this process pertains to the precise separation and extraction of nanowires from the base image. To demonstrate the efficacy of our pre-processing methodology, we have chosen to employ a compact image classification architecture implemented in the PyTorch framework (RRID:SCR 018536) ^36, 37^. The architectural configuration comprises a pair of convolutional layers succeeded by a trio of linear layers. The model structure is shown in Figure 3. The optimization process integrates the cross-entropy loss function with the Adam algorithm. Training was carried out for 100 iterations until loss saturation was achieved. The model uses a standard convolution approach, which has been used successfully to identify the class of images from popular datasets such as the CIFAR-10 ^38^.

**Figure 4.**
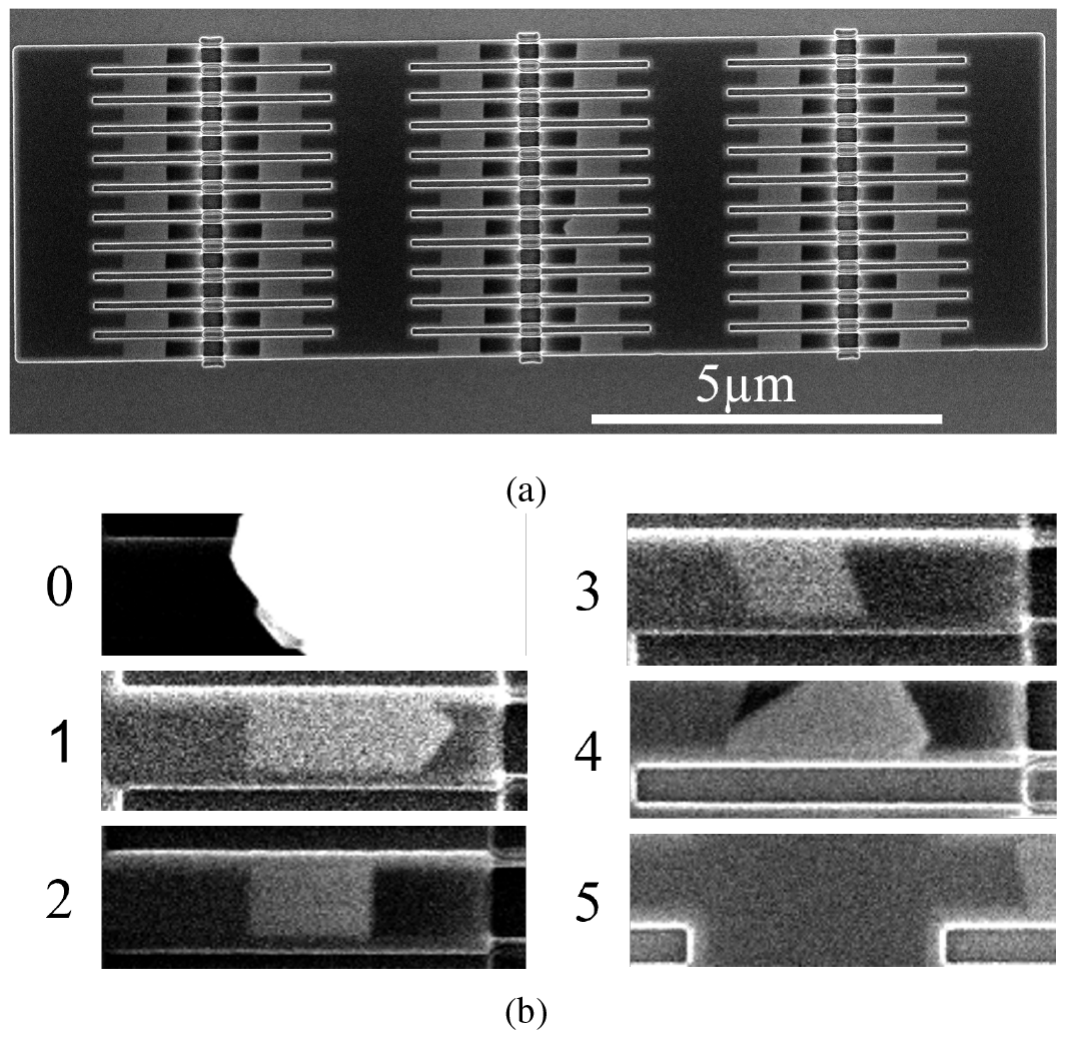
Scanning electron microscopy (SEM) images of two 11 × 6 nanowire arrays. **a**) 210nm wide wires in a template parallel to a ⟨1 1 1⟩ in-plane direction. The wires appear as light grey segments. **b**) image portions representing each of the classes as cut by Algorithm 1.

### Dataset

The dataset consists of 240 images containing arrays of 66 nanowires such as that presented in Figure 4a ^39^, therefore, a total of 15840 nanowire nucleation sites are available in the dataset. 60% of the images contain arrays for the growth of wires in the “parallel” configuration and the remaining 40% consists of images of arrays in the “tilted” configuration. The wires were grown using TASE ^14, 19, 23^. The images were captured with the electron beam of a FEI Helios NanoLab 450S in SEM mode. The arrays are made of different nanowire widths, with four sizes present: 70nm, 140nm, 210nm, 280nm, and in orientation with respect to the in-plane ⟨1 1 1⟩ directions. As the Si crystal determines the seed surface and the final facet orientation, changing the template orientation will produce a tilted seed and end facet ( Figure 4b, class 3). SEM images are used for quality control in nanofabrication, as they allow for high magnification and survey speeds. Traditionally, once an SEM image is taken, the operator determines whether the nanowires contained in it are defective. We have surveyed the nanowire arrays and labelled them to produce the dataset used in this paper ^39^.

The examples shown in Figure 4b provide an idea of what each class looks like. The wires were labelled using LabelMe ^40^, with four classes used to capture the differences between the wires grown along a *⟨*1 1 1 *⟩* direction and those grown tilted away from it ( Table I). This produced the first four classes: ”Wire Parallel Perfect”, ”Wire Parallel Defect”, ”Wire Tilted Perfect”, and ”Wire Tilted Defect”. They represent 49.7%, 2.4%, 21.6%, and 1.6% of the dataset, respectively. Therefore, an imbalance exists between the number of defective and perfect wires, with the latter greatly outnumbering the former and an overall prevalence of the ”Wire Parallel Perfect” class in the dataset.

**Table I.** Class encodings.

		Wire Parallel		Wire Tilted		
Class	Parasitic	Defect	Perfect	Defect	Perfect	Null
Encoding	0	1	2	3	4	5

A fifth class is necessary because of the presence of parasitic growth on the surface of some arrays. Parasitic growth occurs on the rare occasion when III-V material nucleates on the walls of the template SiO _2_. As there is no template to contain the growth of this parasitic crystal, it expands faster, covering a large number of templates.

A ”Null” class is introduced to take into account the possibility that the pre-processing algorithm could have sliced the image incorrectly. This can be due to a number of factors, such as a tilt of a few degrees of the structure in the image or the influence of a large parasitic growth crystal in the cutting of the array contours. The percentage of ”Null” class images in the dataset was calculated at 12.6%.

### Experiment set-up

**Figure 5.**
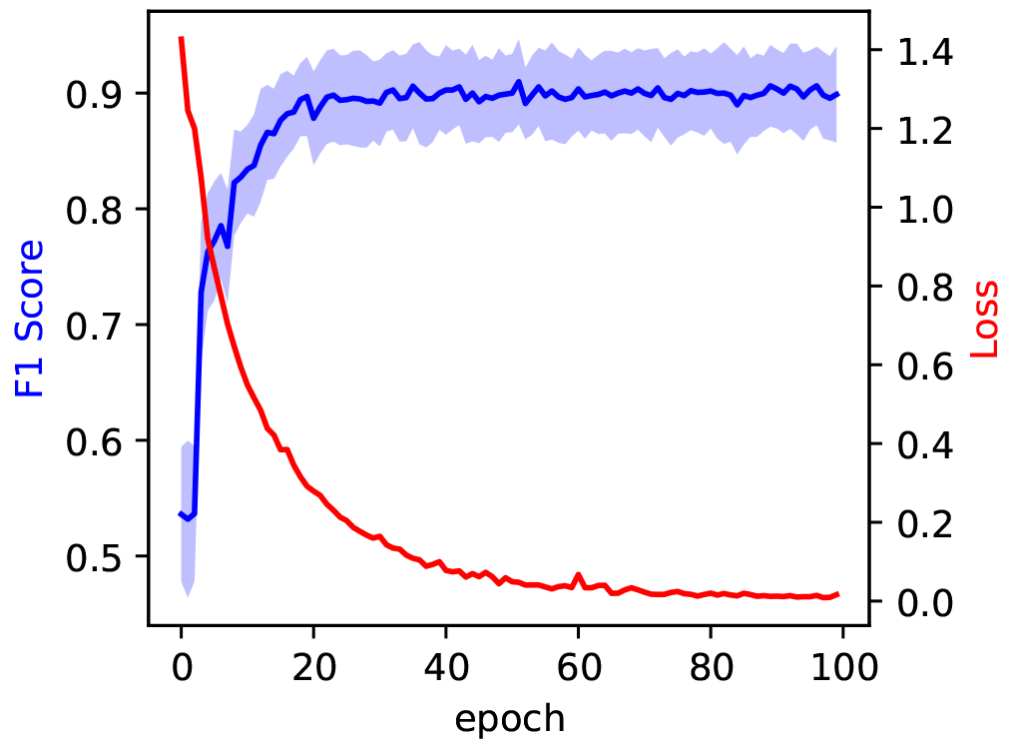
In blue, the evolution of the F1 score during model training, measured on the test set at each epoch. The blue shaded area corresponds to the 97% confidence interval. The evolution of the training loss is measured on the training set at each epoch and reported in red.

We divide the data set into training and validation sets with a 70/30 split. We trained the model for 100 epochs. The training loss is shown to saturate around 100 epochs in Figure 5, indicating the successful training of the model. Two metrics, precision and recall, are used to evaluate the performance of a classifier when encountering new data that it was not presented with, during the training stage. Precision and recall refer to the ability of the model to correctly identify an item in the test set as belonging to a class. Precision is the ratio of true positives to the sum of true and false positives. Recall, on the other hand, is defined as the ratio of true positives to the sum of true positives and false negatives. Precision and recall can be combined into the F1 score, a metric that gives an overall assessment of the quality of the model. The F1 score of the best model was recorded on the test set at 0.91 *±* 0.04 (97% confidence).

**Figure 6.**
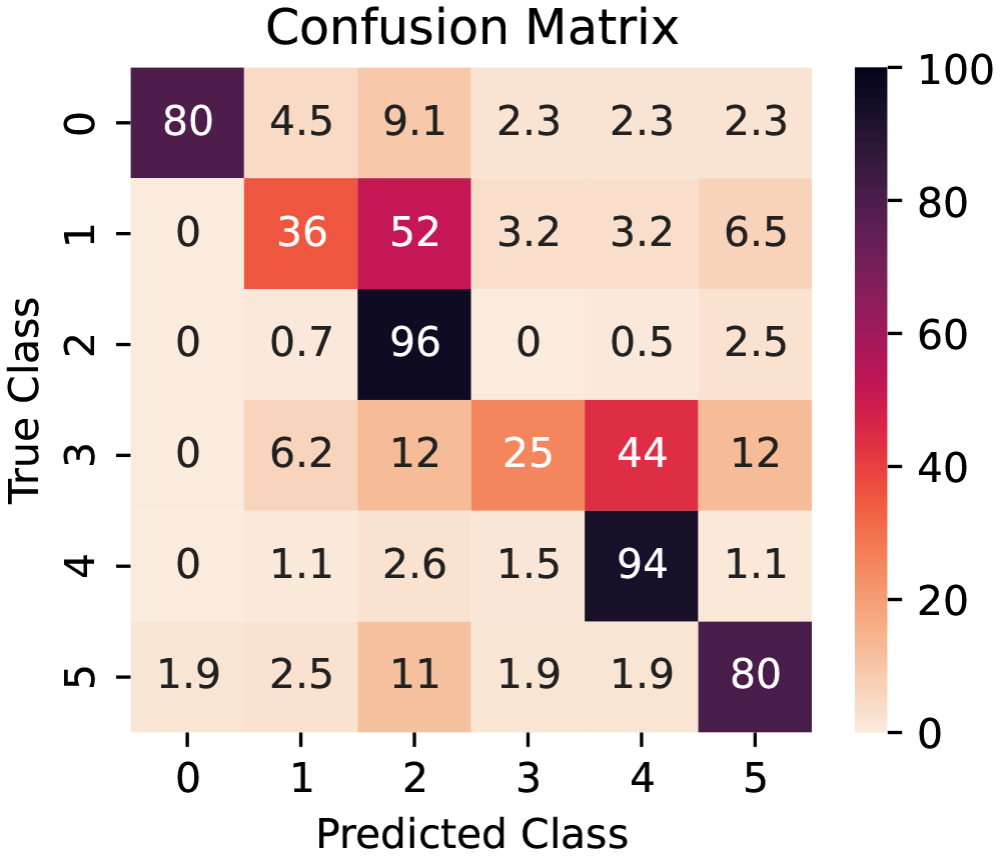
Confusion matrix for the model (from test set). The values are row-normalized and presented as percentages.

In some circumstances, such as when an imbalanced dataset is employed, a single number metric such as the F1 score can, however, give a misleading or incomplete ”idea” of the model’s performance. In such a situation, per-class metrics provide greater insight into a model’s performance. The confusion matrix in Figure 6 summarizes the per-class performance of the model. The distribution of the predictions for each class is shown on each row. The data is presented as percentages: as such, the diagonal (top left to bottom right) also indicates the percentage recall of each class.

## Results

The model shows outstanding performance in nanowire classification: achieving a total F1 score of 0.91 *±* 0.04. This surpasses similar methods for nanowire classification by roughly 0.1 ^30^ while using a more compact model with lower computational costs. The model shows excellent overall performance, exemplified by the high F1 score of 0.91 *±* 0.04 obtained from training on an original dataset of only 240 images. The F1 Score saturates early in the training process, after around 30 epochs, as is evident by the blue curve in Figure 5.

The model’s performance is different depending on the class it is trying to predict. The Recalls for each class are represented in the diagonal elements of the confusion matrix in Figure 6 and illustrate how the model performs when distinguishing between different classes. It can easily distinguish between non-defective parallel and tilted wires and reliably identify wrongly cut images containing partial III-V nanowires (non-defective class) or parasitic crystals. However, the model struggles to correctly classify defective wires in the test data set, with percentages of 36% and 5% of correctly identified parallel and tilted wires (classes 1 and 3). Conversely, defective wires are more likely to be classified as non-defective (52% and 44%). This is due to a combination of factors: there is a scarcity of defective wires in the data set, totaling less than 5% of the data, and growth failures can occur in more than one configuration, often with a small deviation from the desired shape creating the defect. Therefore, the preprocessing algorithm is not a contributing factor in this metric.

## Conclusions

In this work, we have shown a novel algorithm to separate individual nanowires from nanowire arrays to create a general model to classify nanowires as defective and non-defective and differentiate between different growth configurations. We achieved an F1 score of 0.91 in the test set, and the recall and confusion matrix have shown a high competence in the distinction between parallel and tilted wires and parasitic growth.

Expanding the initial data set with more SEM images will likely help overcome the difficulties in classifying defective wires. We expect this expansion to not only aid in recognition of defective wires but also to be able to correctly classify tase wires grown with other crystalline orientations, given the current model’s ability to distinguish between parallel and tilted wires. Furthermore, in an industrial application where the tilt of the sample and the contrast and brightness are more homogeneous than the ones in the present dataset, the prevalence of the Null class is expected to be reduced. A more complex machine learning model could achieve better performance; however, our preprocessing approach has proven capable of creating an input dataset well suited for a lightweight model.

Our manual segmentation approach is particularly powerful, as it relies on precise image gathering, easily achievable with SEM instrumentation, and can be further enhanced by a data acquisition algorithm. The preprocessing algorithm has proven to be a robust way to extract single nanowire images, reducing the complexity of the machine learning model’s task. This produced a small, quick, and efficient model that has achieved a high degree of accuracy.

## Ethics and consent

Ethical approval and consent were not required.

## Data Availability

The Dataset used in this study is available at https://doi.org/10.5281/zenodo.10204018 ^39^. This project contains the following data: SEM Images of Arrays of III-V Nanowires with Labelled Data.zip (.zip folder containing all SEM images used). Data are available under the terms of the Creative Commons Attribution 4.0 International license (CC-BY 4.0).

## Software availability

The software created in this project is available at: https://doi.org/10.5281/zenodo.10255657 ^32^ under the terms of the Creative Commons Attribution 4.0 International license (CC-BY 4.0).
