# Supplementary material for: Machine Learning Inspired Nanowire Classification Method based on Nanowire Array Scanning Electron Microscope Images
Source: Open Res Eur. 2024 Jun 28;4:43. Originally published 2024 Mar 1. [Version 2] doi: 10.12688/openreseurope.16696.2 (PMC11217720; doi:10.12688/openreseurope.16696.2)
Supplement: Peer review history

# Reviewer report: Reviewer response for version 2

**DOI:** 10.21956/openreseurope.19539.r41761

**Published:** 2024-07-08

**Authors:** Chen Luo

**Affiliations:** 1 Fudan University, Shanghai, China

The authors responded to my questions and the article looks much better.

Is the study design appropriate and does the work have academic merit?

Yes

Is the work clearly and accurately presented and does it cite the current literature?

Yes

If applicable, is the statistical analysis and its interpretation appropriate?

Partly

Are all the source data underlying the results available to ensure full reproducibility?

Partly

Are the conclusions drawn adequately supported by the results?

Partly

Are sufficient details of methods and analysis provided to allow replication by others?

Partly

Reviewer Expertise:

characterization, Semiconductor device, machine learning

I confirm that I have read this submission and believe that I have an appropriate level of expertise to confirm that it is of an acceptable scientific standard.

# Reviewer report: Reviewer response for version 2

**DOI:** 10.21956/openreseurope.19539.r41763

**Published:** 2024-07-01

**Authors:** Jin Chang

**Affiliations:** 1 Delft University of Technology, Delft, Netherlands Antilles

After revision, the current version is more suitable for acceptance.

Is the study design appropriate and does the work have academic merit?

Yes

Is the work clearly and accurately presented and does it cite the current literature?

Partly

If applicable, is the statistical analysis and its interpretation appropriate?

Partly

Are all the source data underlying the results available to ensure full reproducibility?

Partly

Are the conclusions drawn adequately supported by the results?

Yes

Are sufficient details of methods and analysis provided to allow replication by others?

Yes

Reviewer Expertise:

quantum optics, superconducing nanowire single-photon detectors, integrated photonics, and optomechanics.

I confirm that I have read this submission and believe that I have an appropriate level of expertise to confirm that it is of an acceptable scientific standard.

# Reviewer report: Reviewer response for version 1

**DOI:** 10.21956/openreseurope.18032.r39757

**Published:** 2024-05-25

**Authors:** Ning Yu

**Affiliations:** 1 Stanford University, Stanford, California, USA; 2 UC Riverside, Riverside, California, USA

In this article, the authors presented an innovative way to isolate and distinguish individual nanowires within nanowire arrays with machine learning. The ability to identify nanowires from SEM images, which usually contains high noise, could significantly improve the efficiency of characterization. This work is valuable if could be generalized to guide researchers in academia and industry.

 In the meanwhile, I think there are aspects that this manuscript could improve. Some points the authors wished to make were vague. For example, “metal interconnects offer their own challenges at such small scales” could be more informative by listing a few of those challenges.

 In array splitting, the authors stated the algorithm can work effectively with small degrees of tilt but may fail with larger tilts. Could the authors provide an estimation of the tilt range that could work?

 I am also curious about if the performance of this methodology differs with different size of nanowires and if SEM images of different magnifications could be analyzed at the same time.

Is the study design appropriate and does the work have academic merit?

Yes

Is the work clearly and accurately presented and does it cite the current literature?

Yes

If applicable, is the statistical analysis and its interpretation appropriate?

Yes

Are all the source data underlying the results available to ensure full reproducibility?

Yes

Are the conclusions drawn adequately supported by the results?

Yes

Are sufficient details of methods and analysis provided to allow replication by others?

Yes

Reviewer Expertise:

nanomaterials, catalysis, energy, optics

I confirm that I have read this submission and believe that I have an appropriate level of expertise to confirm that it is of an acceptable scientific standard, however I have significant reservations, as outlined above.

# Response

**Published:** 2024-06-13

**Authors:** Vihar Georgiev

**Affiliations:** 21828, UK

Reply to 1:  Added aspect ratio limit and cross talk as examples. The reference goes in greater detail about these.

 Reply to 2: 5 degrees of tilt in either direction or more starts giving problems to the algorithm, as empirically observed.

 ​​​​​​​Reply to 3:  ​​​​​​​​​​​​​​In theory yes. The main prerequisites for the splitting algorithm are that it assumes the geometry of the wire arrays, but if provided pictures of TASE nanowires the algorithm should be able to recognise them as such on the basis of the dataset. Unfortunately, as seen with the current dataset, it will likely not be able to recognise them ad defective if they are. Further development is necessary in this direction.

# Reviewer report: Reviewer response for version 1

**DOI:** 10.21956/openreseurope.18032.r38958

**Published:** 2024-05-25

**Authors:** Jin Chang

**Affiliations:** 1 Delft University of Technology, Delft, Netherlands Antilles

**Review Report**

 The authors Enrico et.al demonstrated a study and methodology for the classification of nanowires within scanning electron microscope (SEM) images. The study employs image manipulation techniques and machine learning algorithms to accurately identify and categorize individual nanowires in SEM images depicting nanowire arrays. The methodology is demonstrated on a dataset comprising 240 III-V nanowire arrays grown on silicon substrates. Results indicate good precision and recall in distinguishing between different wire configurations and detecting parasitic crystals, with an average F1 score of 0.91. The paper emphasizes the importance of precise image gathering, robust preprocessing algorithms, and image processing paradigms to mitigate the influence of noise on nanowire classification. The findings highlight the viability of the proposed technique for academic research and potential practical commercial applications in the field of nanomaterials and microscopy imaging.  Generally, the research topic is well-defined, supported by ample data, and analyzed appropriately. Below, I will outline several questions. If the authors address these questions and refine the manuscript accordingly, it will enhance the quality of the manuscript and render it suitable for acceptance.

*Questions 1-4 are mainly about the background/motivation, and 5-9 focus on the methodology.*

 (1) In the introduction, the authors present the challenge of connecting transistors over short distances with III-V photonics components. This goal is ambitious; however, the references cited do not adequately represent the progress made in this area. Reference 6 primarily focuses on Photonic Integrated Circuits (PICs) rather than the interconnection of CMOS elements with photonics components, and reference 5 dates back to 2000. Are there any recent studies demonstrating advancements in connecting transistors with III-V elements? If so, they should be cited appropriately to strengthen the motivation behind this work.

 (2) The author mentioned that “…selected growth conditions that stabilize a single {1 1 1} facet as a growth front perpendicular to the wafer surface18. From this knowledge, it is already possible to classify the wires as perfect from an SEM top-down view of the wire if the seed facet and the end facet are parallel along the growth direction and as defective otherwise.” Understandably, opting for this choice simplifies the subsequent nanowire automatic classification process, as it relies solely on SEM pictures. However, the question arises: in real-world application scenarios, such as interconnecting transistors with III-V nanowires, is this nanowire growth configuration still appropriate for interconnection? If the answer is yes, proper citation would be beneficial. However, if the answer is no, it suggests that the mentioned classification method may not be versatile.

 (3) What are the critical physical properties of the nanowires in this study in terms of future applications? For instance, are factors such as optical loss and emission wavelength significant? Given that the classification relies solely on SEM pictures, there may not be a rigorous connection between morphology and performance. Has the author measured any physical properties of the nanowires post-classification to confirm the relationship between SEM images and actual performance?

 (4) What are the specific III-V materials utilized for the nanowires in this study? And could they be electrically pumped? Because this is important for interconnecting transistors with photonics.

 (5) Regarding the software/code, although the code contains method names and some comments, it lacks comprehensive documentation that explains the purpose of each method, expected inputs, and return values. Adding proper notes to the code would be beneficial.

 (6) It appears that there is no exception-handling function in the code. For instance, in SmartImage.py, there is no implementation of try-except blocks or other error-handling techniques. Consequently, if an error arises during image processing or manipulation, the code may crash without offering informative error messages.

 (7) The dataset utilized for training the machine learning model comprises 240 SEM images of III-V nanowire arrays. Is this quantity adequate, or is it somewhat constrained in terms of size? While the dataset might suffice for the initial development of the model, a larger and more varied dataset could enhance the model's resilience and its ability to generalize to unseen data. A limited dataset size could introduce bias into the model and might fail to capture the complete range of nanowire configurations.

 (8) The code lacks explicit measures to prevent overfitting, such as regularization techniques or early stopping based on validation performance. This omission could result in the model memorizing the training data and subsequently performing poorly when faced with unseen data.

 (9) The code overlooks the potential issue of class imbalance within the dataset. If certain classes are underrepresented, the model may face challenges in effectively learning from them, leading to biased predictions, especially considering that most nanowires are classified as "good."

 If the aforementioned questions can be properly addressed, the manuscript may be considered for acceptance.

Is the study design appropriate and does the work have academic merit?

Yes

Is the work clearly and accurately presented and does it cite the current literature?

Partly

If applicable, is the statistical analysis and its interpretation appropriate?

Partly

Are all the source data underlying the results available to ensure full reproducibility?

Partly

Are the conclusions drawn adequately supported by the results?

Yes

Are sufficient details of methods and analysis provided to allow replication by others?

Yes

Reviewer Expertise:

quantum optics, superconducing nanowire single-photon detectors, integrated photonics, and optomechanics.

I confirm that I have read this submission and believe that I have an appropriate level of expertise to confirm that it is of an acceptable scientific standard, however I have significant reservations, as outlined above.

# Response

**Published:** 2024-06-13

**Authors:** Enrico Brugnolotto

**Affiliations:** University of Glasgow, Glasgow, Scotland, UK

We thank the reviewer for the time taken to read the article and provide useful feedback.

1. Added a reference from 2017
2. Citations about TASE explain the advantages of this fabrication method in integration. The main one is the co-planar self-aligned integration of III-V active photonic elements with silicon waveguides.
3. The advantage of TASE is in it co-planar integration and self-alignment with silicon waveguides, on which ends active III-V photonic components can be grown. Optical loss and emission wavelength can be important, depending on the application, for example due to the use of wavelengths that can be guided losslesly in silicon. While it is possible that this growth approach would not make a large difference for some applications which do no require sophisticated hardware, it stands to reason that the ordered and reliable growth of III-V structure is preferrable to a disorganised growth. It is a simple fact that a greater variety of growth outcomes are directly correlated with a greater variery of performance outcomes. This situation is the one that is prevalent in growth in templates with methods like TASE and LART. While performance is important in industry, stardandization is crucial, and having different nanowire lengths and internal compositions (in the case of ternary materials) is not conductive to the type of automated processes in industry. Before the question of performance is asked, a simpler question is that of contacting. If the various material layers of a heterostructured p-i-n detectors all have different lengths in each wire contacting it requires a wire-by-wire adjustment of the lithography mask defining the metal contacts. This is obviously highly sub-optimal for large scale integration densities.
4. They are InP and InGaAs. A device based on these materials and created with TASE was tested with electrical pumping in reference 15.
5. Comments were added in the code to improve readability.
6. It will not be possible to revise the code substantially due to the project having ended.
7. While it is true that the dataset does not represent the entirety of the nanowire grown it is still a collection of 15k+ growth sites. The main issue with the dataset in terms of machine learning is that the fraction of defective wires is small compared to the total. This, coupled with the simple fact that the defective wires fail in different ways, makes those two classes have poor recalls. Unfortunately due to the project being already over,  it will not be possible to supplement additional images to the existing dataset that, in our opinions, remains a large collection of nanowires imaged with SEM and is freely available for use even outside of the scope of the current publication.
8. It will not be possible to revise the code substantially due to the project having ended.
9. This is true and the bias towards “perfect” wires is seen in the recall, however the model has high performance distinguishing other classes, and not just markedly different ones such as “Parasitic” or Null” from perfect wires but also between tilted and perpendicular wires. It will not be possible to revise the code substantially due to the project having ended. However, it is publicly available, and can be used and modified by anyone free of charge under the CC BY 4.0 licence. Both improvements to the existing code and new approach in the use of the dataset, maybe in conjunction with other datasets, by other researchers is something we are excited about.

# Response

**Published:** 2024-06-13

**Authors:** Vihar Georgiev

**Affiliations:** 21828, UK

Repy to point 1:  Added a reference from 2017, an exhaustive review

 Reply to point 2:    There are citations about TASE that explain the advantages of this fabrication method in integration. The main one is co-planar self-aligned integration of III-V active photonic elements with silicon waveguides.

 Reply to point 3:    The advantage of TASE is in it co-planar integration and self-alignment with silicon waveguides, on which ends active III-V photonic components can be grown. Optical loss and emission wavelength can be important, depending on the application, for example due to the use of wavelengths that can be guided losslesly in silicon. While it is possible that this growth approach would not make a large difference for some applications which do no require sophisticated hardware, it stands to reason that the ordered and reliable growth of III-V structure is preferrable to a disorganised growth. It is a simple fact that a greater variety of growth outcomes are directly correlated with a greater variery of performance outcomes. This situation is the one that is prevalent in growth in templates with methods like TASE and LART. While performance is important in industry, stardandization is crucial, and having different nanowire lengths and internal compositions (in the case of ternary materials) is not conductive to the type of automated processes in industry. Before the question of performance is asked, a simpler question is that of contacting. If the various material layers of a heterostructured p-i-n detectors all have different lengths in each wire contacting it requires a wire-by-wire adjustment of the lithography mask defining the metal contacts. This is obviously highly sub-optimal for large scale integration densities.

 Reply to point 4:  They are InP and InGaAs. A device based on these materials and created with TASE was tested with electrical pumping in reference 15.

 Reply to point 5:    ​​​​​​​Comments were added in the code to improve readability.

 Reply to points 6 & 7:  ​​​​​​​While it is true that the dataset does not represent the entirety of the nanowire grown it is still a collection of 15k+ growth sites. The main issue with the dataset in terms of machine learning is that the fraction of defective wires is small compared to the total. This, coupled with the simple fact that the defective wires fail in different ways, makes those two classes have poor recalls. Unfortunately due to the project being already over,  it will not be possible to supplement additional images to the existing dataset that, in our opinions, remains a large collection of nanowires imaged with SEM and is freely available for use even outside of the scope of the current publication.

 ​​​​​​​Reply to points 8 & 9:   ​​​​​​​This is true and the bias towards “perfect” wires is seen in the recall, however the model has high performance distinguishing other classes, and not just markedly different ones such as “Parasitic” or Null” from perfect wires but also between tilted and perpendicular wires. It will not be possible to revise the code substantially due to the project having ended. However, it is publicly available, and can be used and modified by anyone free of charge under the CC BY 4.0 licence. Both improvements to the existing code and new approach in the use of the dataset, maybe in conjunction with other datasets, by other researchers is something we are excited about.

# Reviewer report: Reviewer response for version 1

**DOI:** 10.21956/openreseurope.18032.r40206

**Published:** 2024-05-22

**Authors:** Chen Luo

**Affiliations:** 1 Fudan University, Shanghai, China

The article introduces an innovative classification methodology to identify nanowires within SEM images. The topic is interesting but the article is not well organized. There are some problems that need to be solved.

 1. Authors compared STEM and SEM methods in the introduction. The STEM method allows for the visualization of defects, while SEM determines whether they are parallel to the growth direction while stabilizing {1 1 1} facet as a growth front perpendicular to the wafer surface. Is there a possibility that even if the growth is parallel, there will still be defects? Or how to define “parallelism” from the perspective of SEM top down view? If the nanowires are not uniformly straight, how many degrees is considered parallel?  

 2. In Figure 3, each part in the SEM image needs to be labeled, such as which part is the nanowire and which part is the substrate. It is difficult to understand, for example, please show the difference in morphology between perfect nanowires and defective nanowires in the top down view.  

 3. Figure 4 mentioned first in the main text, followed by Figure 3.  

 4. All SEM images in the text should have a scale bar.  

 5. Figure 1 is too small, it would be best to show the images after each operation, such as splitting and after removing noise.

Is the study design appropriate and does the work have academic merit?

Yes

Is the work clearly and accurately presented and does it cite the current literature?

Yes

If applicable, is the statistical analysis and its interpretation appropriate?

Partly

Are all the source data underlying the results available to ensure full reproducibility?

Partly

Are the conclusions drawn adequately supported by the results?

Partly

Are sufficient details of methods and analysis provided to allow replication by others?

Partly

Reviewer Expertise:

characterization, Semiconductor device, machine learning

I confirm that I have read this submission and believe that I have an appropriate level of expertise to confirm that it is of an acceptable scientific standard, however I have significant reservations, as outlined above.

# Response

**Published:** 2024-06-13

**Authors:** Enrico Brugnolotto

**Affiliations:** University of Glasgow, Glasgow, Scotland, UK

We thank the reviewer for their time and precious feedback.

1. ​​​​​​​​​​​​​​Yes, it is in theory possible, and it mostly depends on what defects are considered “acceptable” for the purpose of device fabrication. This is mainly a growth study, and the yields are mechanical, as in, we try to stabilise a certain growth type and achieve high yield of geometrically similar wires. As such no device characterisation was carried out. Further reading supporting the hypothesis that the internal composition is reproducible and can be inferred by the external shape of the wires is in reference 38. The nanowires are uniformly “straight” as in they are constrained to develop in the same direction by the template they grow in. I believe what you are asking is “how many degrees of difference can there be between the vector defining the plane of the seed and thant defining the plane of the end facet?” in reality small tilts of a handful of degrees (up to 5) can be seen as a stress relaxing avenue. This tilt originates in the III-V lattice in the very few first atomic bi-layers after nucleation. It is not indicative, however, of any defects in the wire and is very difficult to spot from a top down view of the wire. Real indicators of defects are end-interfaces that are composed of multiple crystalline facets, or are at high angles compared to the seed facet as shown later on in the class breakdown.
2. The differences between perfect and defective nanowires are shown in Figure 4.
3. It is mentioned first, but addressed second.
4. Figure 4 provides a scalebar which allows the reader to understand the scale of the nanowires. Of course, scale bars are present in all images in the freely available dataset allowing them to be reused in other project with the knowledge of the scale of the structures pictured.
5. ​​​​​​​Example of images after splitting are available in Figure 4(B), the denoised image does not visibly differ from those shown at this magnification level.

# Response

**Published:** 2024-06-13

**Authors:** Vihar Georgiev

**Affiliations:** 21828, UK

Reply to Point 1:  Yes, it is in theory possible, and it mostly depends on what defects are considered “acceptable” for the purpose of device fabrication. This is mainly a growth study, and the yields are mechanical, as in, we try to stabilise a certain growth type and achieve high yield of geometrically similar wires. As such no device characterisation was carried out. Further reading supporting the hypothesis that the internal composition is reproducible and can be inferred by the external shape of the wires is in reference 38. The nanowires are uniformly “straight” as in they are constrained to develop in the same direction by the template they grow in. I believe what you are asking is “how many degrees of difference can there be between the vector defining the plane of the seed and thant defining the plane of the end facet?” in reality small tilts of a handful of degrees (up to 5) can be seen as a stress relaxing avenue. This tilt originates in the III-V lattice in the very few first atomic bi-layers after nucleation. It is not indicative, however, of any defects in the wire and is very difficult to spot from a top down view of the wire. Real indicators of defects are end-interfaces that are composed of multiple crystalline facets, or are at high angles compared to the seed facet as shown later on in the class breakdown.

 Reply to Point 2:  The differences between perfect and defective nanowires are clearly shown in Figure 4.

 Reply to Point 3:  ​​​​​​​It is mentioned first, but addressed second.

 Reply to Point 4: ​​​​​​​Figure 4 provides a scalebar which allows the reader to understand the scale of the nanowires. Of course, scale bars are present in all images in the freely available dataset allowing them to be reused in other project with the knowledge of the scale of the structures pictured.

 ​​​​​​​Repy to Point 5:  ​​​​​​​Example of images after splitting are available in Figure 4(B), the denoised image does not visibly differ from those shown at this magnification level.

# Reviewer report: Reviewer response for version 1

**DOI:** 10.21956/openreseurope.18032.r38954

**Published:** 2024-05-15

**Authors:** Nasser Mohieddin Abukhdeir

**Affiliations:** 1 University of Waterloo, Waterloo, Ontario, Canada

The manuscript presents a computational method to classify SEM (or other microscopy) images of nanowire structures through the combination of a pre-processing image segmentation method followed by a convolutional neural network which has been trained for a sample qualitative classification. While the presented method is likely novel and both source data/code have been made available, the novelty and generality of the presented method is not well-supported in the manuscript. A significant literature of past image analysis and processing methods focused on nanowires has not been well-reviewed or cited, which does not provide the reader/reviewer with the basic context of this research within the current archival literature. Furthermore, apart from minimally showing that the method functions for a very limited set of experimental data, there are no results presented showing its application to other relevant experimental data sets and demonstration of its utility/impact to better understand these results. Unfortunately I do not think this manuscript meets the minimum standards for indexing.

Is the study design appropriate and does the work have academic merit?

Partly

Is the work clearly and accurately presented and does it cite the current literature?

Partly

If applicable, is the statistical analysis and its interpretation appropriate?

Yes

Are all the source data underlying the results available to ensure full reproducibility?

Yes

Are the conclusions drawn adequately supported by the results?

No

Are sufficient details of methods and analysis provided to allow replication by others?

Partly

Reviewer Expertise:

NA

I confirm that I have read this submission and believe that I have an appropriate level of expertise to state that I do not consider it to be of an acceptable scientific standard, for reasons outlined above.

# Response

**Published:** 2024-06-13

**Authors:** Vihar Georgiev

**Affiliations:** 21828, UK

​​​​​​While it is true that the method has no aspiration to classify any nanowire grown with any technique, its application is in the context of TASE-grown nanowires. It is already sufficiently accurate in distinguishing between two different TASE nanowire configurations and parasitic crystals. Further expansion of the dataset and code to include more
